# Oxazolidine Nitroxide Transformation in a Coordination Sphere of the Ln^3+^ Ions

**DOI:** 10.3390/molecules27051626

**Published:** 2022-03-01

**Authors:** Philippe Rey, Anton I. Smolentsev, Kira E. Vostrikova

**Affiliations:** Nikolaev Institute of Inorganic Chemistry, Siberian Branch, Russian Academy of Sciences, 630090 Novosibirsk, Russia; philippe.rey@etu.univ-grenoble-alpes.fr (P.R.); smolentsev@ngs.ru (A.I.S.)

**Keywords:** lanthanide complexes, stable organic radical, tripodal ligands, oxazolidine nitroxyl radical, disproportionation, catalytic hydrolysis

## Abstract

Upon the interaction of the hydrated lanthanide(III) salts found in acetonitrile solution with a tripodal paramagnetic compound, 4,4-dimethyl-2,2-bis(pyridin-2-yl)-1,3-oxazolidine-3-oxyl (Rad), functionalized by two pyridyl groups, three neutral, structurally characterized complexes with diamagnetic polydentate ligands—[Dy(RadH)(hbpm)Cl_2_], [Yb_2_(ipapm)_2_(NO_3_)_4_], and [Ce_2_(ipapm)_2_(NO_3_)_4_(EtOAc)_2_]—were obtained. These coordination compounds are minor uncolored crystalline products, which were formed in a reaction mixture due to the Rad transformation in a lanthanide coordination sphere, wherein the processes of its simultaneous disproportionation, hydrolysis, and condensation proceed differently than in the absence of Ln ions. The latter fact was confirmed by the formation of the structurally characterized product of the oxazolidine nitroxide transformation during its crystallization in toluene solution. Such a conversion in the presence of 4f elements ions is unique since no similar phenomenon was observed during the synthesis of the 3*d*-metal complexes with Rad.

## 1. Introduction

For the design of molecular materials with specified physical properties, it is usually necessary to obtain compounds with a certain symmetry in the coordination polyhedron. This requires the polydentate ligands to possess a predictable coordination manner, along with stereochemical rigidity and satisfactory steric hindrance. Aside from this, the organic ligands corresponding with these requests must be stable during the synthesis of coordination compounds in atmospheric conditions if we want to determine their application in materials science.

The design, synthesis, and study of molecular magnetic materials are actively developing areas [1,2]. Zero-dimensional (0D) magnetic compounds are especially attractive because it is much easier to theoretically model the magnetic behavior for such heterospin systems. Of particular interest are the single-ion complexes of paramagnetic metals with stable organic radicals, the spin centers of which are directly bound to a metal center, thus providing strong exchange interactions between the spins [3,4,5].

**Scheme 1:**
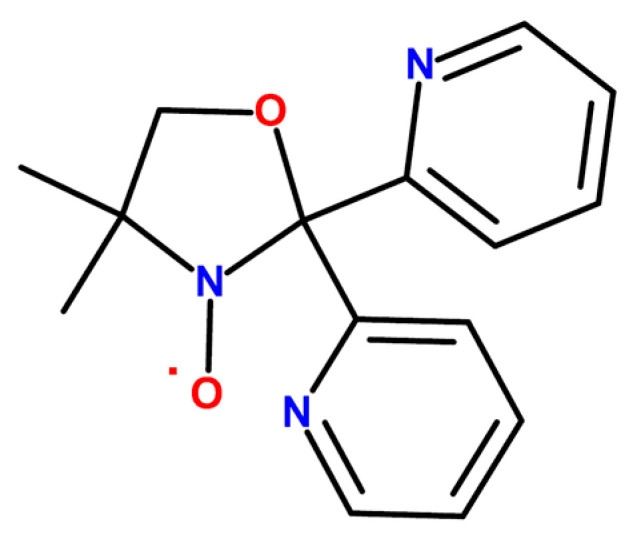
Tridentate radical—4,4-dimethyl-2,2-bis(pyridin-2-yl)-1,3-oxazolidine-3-oxyl (Rad).

In connection with the above, the functionalized oxazolidine nitroxyl radical 4,4-dimethyl-2,2-bis(pyridin-2-yl)-1,3-oxazolidine-3-oxyl (Rad; Scheme 1) is an attractive ligand for the design of complexes having axial point symmetry (D_3h_) that could ensure the slow relaxation of magnetization [6,7,8], and therefore, magnetic bistability. The coordination ability of Rad has been tested on the 3*d* metal-ions (M^3d^) [9,10,11,12,13,14,15]. The total majority of Rad-M^3d^ complexes contains two paramagnetic ligands. The diradical compound, [Co^II^Rad_2_](NO_3_)_2_, as shown by dynamic squid magnetometry, displays field-induced slow magnetic relaxation, which indicates that this complex is a single-ion quantum magnet (SIM) [11]. The monoradical complexes of Rad were only obtained for Ni^2+^ [14].

Quite recently, we have firstly prepared several monoradical complexes of Ln(III), [LnRad(NO_3_)_3_] [16]. The obtained value of 23 cm^−1^ for the antiferromagnetic metal-radical coupling in [GdRad(NO_3_)_3_], established from the magnetic and EPR data, is outstanding for the complexes of 4f-elements with nitroxyl radicals. The terbium congener displays frequency-dependent, out-of-phase signals in the zero field, designating single-molecule magnetic behavior. In a just-published communication, the two diradical complexes, [LnRad_2_(CF_3_SO_3_)_3_], Ln = Dy and Eu, were reported [17].

During the synthesis of diradical compounds, we have observed that, along with the formation of the major products, a small amount of uncolored complexes were also obtained, depending on synthetic conditions. In this case, part of the tripodal radical undergoes a transformation associated both with the action of the reaction medium and with the influence of the Ln-center, since a significant rearrangement of the paramagnetic ligand occurs in the coordination sphere of the latter.

In this paper, we present the three structurally characterized binuclear complexes of the trivalent Ce, Dy, and Yb, formed due to the transformation of the tripodal oxazolidine nitroxyl radical upon complexation, and compare them with a molecular compound, which was obtained during the recrystallization process of the paramagnetic ligand. In addition, we present previously unknown crystal structures for the radical and its diamagnetic precursor.

## 2. Results and Discussion

A family of the diradical complexes, [M^3d^Rad_2_]^2+^, with different counterions, have previously been studied [9,10,11,12,13,14,15,18]. It should be especially noted that neither hydrolysis nor another transformation of the starting paramagnetic ligand were communicated in the papers devoted to the Rad complexation by M^3d^ ions. However, in the case of the Ln^3+^ ions—which are known for their catalytic properties towards organic entities, resulting in the breaking of covalent bonds [19,20,21]—extra precautions are needed. In addition, one should pay attention to air humidity and the reaction conditions to avoid by-product formation.

### 2.1. Influence of Moisture and Acidity

**Scheme 2:**
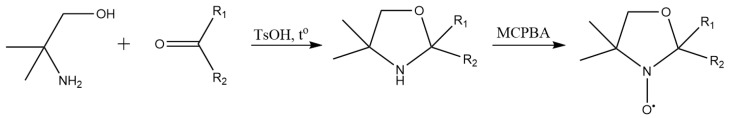
Two-stage preparation of the oxazolidine nitroxyl radicals for Rad R_1_ = R_2_ = 2-pyridyl.

**Scheme 3:**
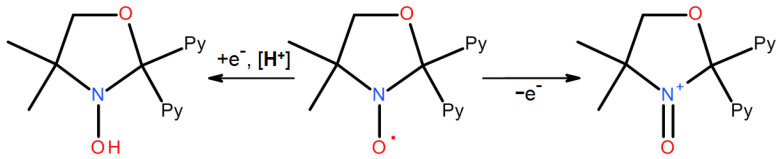
Disproportionation of Rad, center; RadH, left; oxoammonium cation, right.

**Scheme 4:**
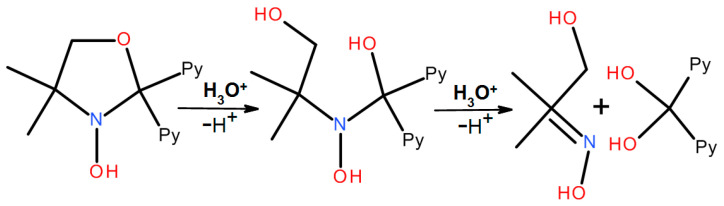
The hydrolysis of RadH (the reduced form of Rad) leading to *hbpmH* (last compound).

The nitroxyl radicals of the oxazolidine type are usually obtained through the oxidation of a diamagnetic precursor [9,21,22,23], which, in turn, is formed through a condensation reaction under the conditions of acidic catalysis (Scheme 2). The reverse process of oxazolidine ring opening is possible in the presence of water molecules; therefore, the hydrolysis process [24] is also very likely for the oxidized form of the oxazolidine heterocycle. In addition, similar to many redox-active compounds, nitroxyl radicals, in general [25], and oxazolidine nitroxides, in particular, undergo disproportionation processes. The disproportionation of nitroxyl radicals in acidic media involves the oxidation half-reaction to oxoammonium cation and the reduction to hydroxylamine, RadH (Scheme 3) [26,27]. Both disproportionation and hydrolysis are catalyzed by the hydronium ion; the latter process for the RadH can be represented as shown in Scheme 4. It should be noted that strong acid catalysis is required to carry out disproportionation and hydrolysis reactions in the absence of metal ions [28]. Unlike nitroxides, which do not have etheric oxygen in their cycle, oxazolidine-type nitroxides are less resistant to hydrolysis in which the cyclic ring is opening.

### 2.2. Transformations in the Presence of Coordination Metal Ions

#### 2.2.1. Tridentate Ligand Formation

**Figure 1:**
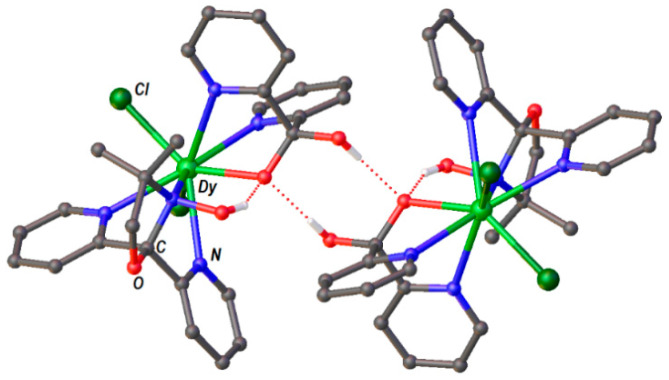
The complex [Dy(RadH)(*hbpm*)Cl_2_] (**1**), dimerized by hydrogen bonding between two *hbpm.* Attached to the carbon atoms, hydrogens and solvate molecules are not shown.

Hydrolysis in the presence of metal ions takes place under mild conditions (it requires a significant catalytic amount of H^+^ and H_2_O), as it is realized in the coordination sphere of ligated complexes [21,29]. Despite the fact that dry acetonitrile was used as a solvent, the amount of water contained in the initial hydrated lanthanide salts was sufficient enough to carry out both the catalytic disproportionation and hydrolysis of Rad coordinated to the Ln-center. In our case, the existence of the simultaneous disproportionation and hydrolysis processes of Rad in a reaction mixture was confirmed by the formation of the mononuclear mixed-ligand neutral complex, [Dy(RadH)(*hbpm*)Cl_2_]CH_3_CN (**1**), where RadH is 3-hydroxy-4,4-dimethyl-2,2-bis(2-pyridyl)oxazolidine, *hbpm*-hydroxybis(2-pyridyl)methanolate (Figure 1). Note that, in the literature, we have not found either free hydroxylamine (RadH) or its complexes. As for the second organic ligand, *hbpm*, apart from **1**, only two Ln-compounds have been structurally characterized: the binuclear complex, [Eu_2_(*dpk*)_2_(*hbpm*)_2_(CF_3_SO_3_)_2_] (*dpk* is 2,2′-dipyridyl ketone), which we recently communicated [17], and one Dy^3+^ polynuclear complex [30]. Unlike the first two compounds, in which the olate O-atom is μ_2_-bridging; in the latter, the same oxygen is μ_3_-coordinated. Crystal data and structure refinement details for compounds **1**–**3** are presented in Table S1 (see Supplementary Materials).

#### 2.2.2. Tetradentate Ligand Formation

**Figure 2:**
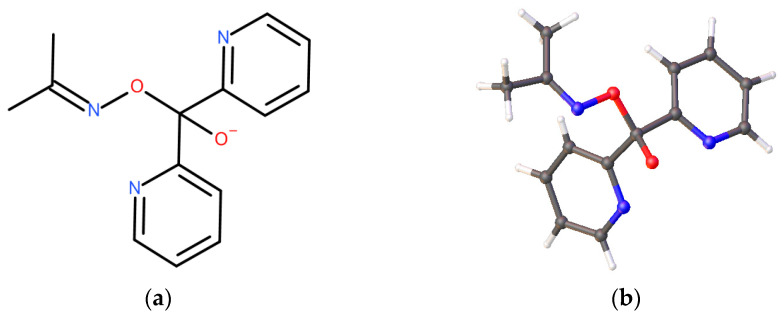
Tetradentate ligand, isopropylideneamino-oxy-bis(2-pyridyl)methanolate (*ipapm)*: (**a**) chemical structure; (**b**) representation from crystal structure.

This subsection is devoted to the in situ formation (Figure S1) of a unique tetradentate ligand, *ipapm* - (isopropylideneamino)oxy-bis(2-pyridyl)methanolate (Figure 2), established by single-crystal X-ray diffraction analysis of binuclear complexes of Ln^3+^: [Yb_2_(*ipapm*)_2_(NO_3_)_4_] (**2**) and [Ce_2_(*ipapm*)_2_(NO_3_)_4_(EtOAc)_2_]·0.5EtOAc (**3**). The *ipapm* ligand is formed during the last stage of the Rad catalytic hydrolysis by self-assembling on the coordination center (Figure S1, see Supplementary Materials). Earlier, a crystal structure was determined for *ipapm*-congener 1,1-bis(1-phenylethylidenaminooxy)ethyl]benzene, which was also synthesized by transition metals ions catalytic condensation [31]. This compound contains a propylidene-amino-oxy synthon related to the (isopropylideneamino)oxy fragment of *ipapm*. The ligands containing RR′**C=N-O-C**(Me)_2_**-O** fragments are also present in the prior studied complexes of transition metals [32,33].

**Figure 3:**
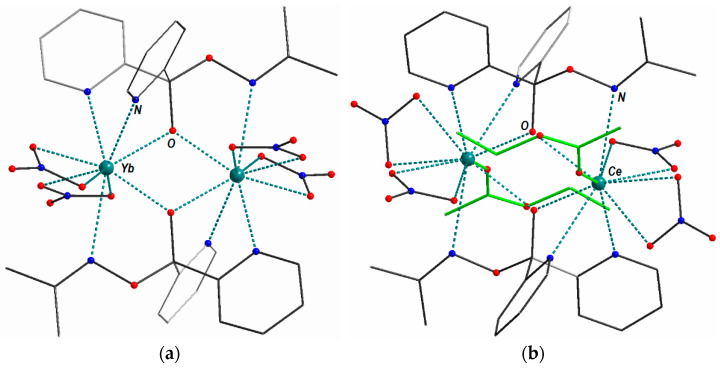
The dimer units of complexes: (**a**) [Yb_2_(*ipapm*)_2_(NO_3_)_4_] (**2**); (**b**) [Ce_2_(*ipapm*)_2_(NO_3_)_4_(EtOAc)_2_] (**3**), EtOAc ligands are highlighted in bright green.

The Ln-dimer species are supported by the two negatively charged *ipapm* (Figure 3). Moreover, each metal center coordinates two bidentate nitrate anions. The smaller Yb^3+^ center in **2** has a coordination number of 9, while the Ce^3+^ ion, being markedly larger, is able to accept an additional donated atom, O, from the ethylacetate ligand (Figure 3b). If the crystals of compound **2** are free of solvate molecules, then complex **3** contains a half molecule of solvated ethylacetate per dimer unit. Based on a thorough analysis of the Cambridge Structural and Reaxys databases, we can state that these two binuclear compounds, supported by two catalytically formed in situ tetradentate *ipapm*-ligands, are unique examples among the complexes, not only of 4f elements but also among other transition metals. Tables S3–S5 contain the selected geometric parameters and numbering schemes for the independent structural parts of **1**–**3**.

#### 2.2.3. Crystallographic Characterization of Rad and Its Diamagnetic Precursor

**Figure 4:**
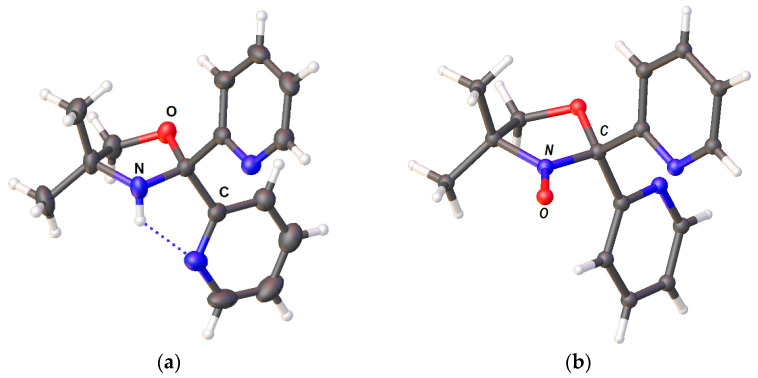
Molecular structures: (**a**) 4,4-dimethyl-2,2-bis(2-pyridyl)oxazolidine (**4**); (**b**) 4-dimethyl-2,2-bis(pyridin-2-yl)-1,3-oxazolidine-3-oxyl (Rad) (**5**).

As shown above, the synthetic protocol for the Rad preparation includes two stages, the first of which is a condensation of di-pyridyl ketone with 2-amino-2-methyl-propan-1-ol, leading to a cyclic amine, 4,4-dimethyl-2,2-bis(2-pyridyl)oxazolidine (**4**). We were able to obtain the single crystals, not only for this precursor but also for the radical, 4,4-dimethyl-2,2-bis(pyridin-2-yl)-1,3-oxazolidine-3-oxyl (**5**). This allowed to characterize of both compounds for the first time. Crystal data and structure refinement details for compounds **4**–**6** are presented in Table S2 (see Supplementary Materials). The molecular structures of **4** and **5** are presented in Figure 4. Tables S6 and S7 involve the selected geometric parameters and numbering schemes for the independent structural parts of **4** and **5**.

#### 2.2.4. The Crystallographic Characterization of 6, a Rad Transformation Product Obtained from the Mother Liquor Remaining after the Recrystallization Process

**Figure 5:**
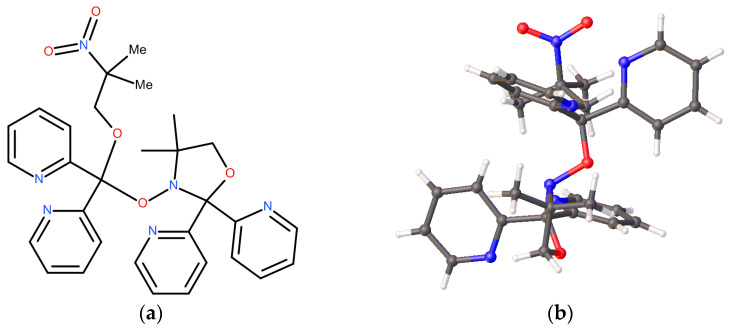
The compound **6**: (**a**) chemical structure; (**b**) molecular structure (side view).

Organic compound (**6**) (Figure 5) is a product of Rad disproportionation, hydrolysis, and condensation, simultaneously occurring in its toluene solution at room temperature for two weeks in the open air. Thus, in the absence of lanthanide ions, the radical transformation also takes place, but it does not occur as dramatically as in the case of the presence of Ln^3+^ ions. Very surprising is the fact that the amino group is oxidized to the nitro group. Most likely, this is a result of the simultaneous occurrence of two processes: disproportionation and action of atmospheric oxygen..

## 3. Experimental Section

### 3.1. Materials and Methods

Lanthanide salts, Ln(NO_3_)_3_·6H_2_O (Ln = Ce, Yb) and DyCl_3_·6H_2_O, were prepared upon the dissolution of the corresponding Ln_2_O_3_ in diluted HNO_3_ or HCL at 50 °C, followed by crystallization during the slow evaporation of the reaction mixture. Solvents of the reagent grade (EKOS-1, Moscow, Russia) were distilled prior to use. 2,2′-Dipyridyl ketone (99%) and 2-amino-2-methyl-1-propanol (99%) (Sigma-Aldrich, Saint Louis, MO, USA) were used as received. The radical, 4,4-dimethyl-2,2-bis(pyridin-2-yl)-1,3-oxazolidine-3-oxyl, was synthesized according to a known procedure [9]. The complexes were synthesized under aerobic conditions. Elemental (C, H, N) analyses were carried out, using standard methods, with a Euro-Vector 3000 analyzer (Eurovector, Redavalle, Italy). The compound structures were visualized, and their drawings were made, with the help of the Diamond 3.0 (Crystal Impact GbR: Bonn, Germany) and OLEX2, Version 1.3. (OlexSys Ltd., Durham University, Durham, UK) software [34].

### 3.2. Single-Crystal X-ray Crystallography Data Collection and Refinement

The single crystals of **1**–**3** were coated with Nujol, mounted on MicroLoops (MiTeGen LLC., Ithaca, NY, USA), and immediately cooled in an N_2_ cold stream to avoid decomposition. The single crystals of **5**–**6** were fixed to the tips of glass fibers with epoxy resin. The data collection was performed at 150 K or room temperature on a Bruker-Nonius X8 Apex (Bruker AXS, Karlsruhe, Germany) 4K CCD diffractometer (graphite monochromatized Mo-Kα radiation, λ = 0.71073 Å). The X-ray data were collected using the standard technique (φ- and ω-scans of narrow frames). Data reduction and multi-scan absorption were carried out using the SADABS (V2014/3, Bruker AXS Inc., Karlsruhe, Germany) [35]. The structures were solved through direct methods and refined by full-matrix least-squares on *F*^2^ using the SHELXL (V2018/3, Göttingen, Germany) software [36]. All non-hydrogen atoms were refined anisotropically. Hydrogen atoms were placed at calculated positions and refined using a riding model. The structure of **6** contained a number of solvent toluene molecules that appeared to be highly disordered, and it was difficult to model their positions reliably. Therefore, the structure was treated via the PLATON/SQUEEZE [37] procedure to remove the contribution of the electron density in the solvent regions from the intensity data, and the solvent-free model from the final refinement was employed. PLATON indicated that the effective volume for solvent molecules was 569 Å^3^, and the electron count per unit cell was 224 e; these parameters were assigned to 4.5 toluene molecules per unit cell, or 1.5 toluene molecules per formula unit. Crystallographic data and refinement details are provided in Tables S1 and S2. The complete crystallographic data for **1**–**6** have been deposited with the Cambridge Crystallographic Data Centre under the reference numbers CCDC 2131650-2131655, respectively. These data can be obtained, free of charge, from CCDC via www.ccdc.cam.ac.uk/structures (accessed on 29 December 2021).

### 3.3. Synthesis

*[Dy(RadH)(hbpm)Cl_2_]CH_3_CN (**1**).* The colorless prismatic crystals of **1** were separated by a filtration of thecooled to RT reaction acetonitrile solution containing DyCl_3_·6H_2_O (38 mg, 0.1 mmol) and Rad (60 mg, 0.222 mmol), which was previously stirred for 30 min at 50 °C. The drying of the compound resulted in an unsolvated complex [Dy(RadH)(hbpm)Cl_2_]. Yield: 5 mg. Anal. calcd. (%) for C_26_H_26_Cl_2_DyN_7_O_4_: C, 42.55; H, 3.6; N, 13.34. Found: C, 42.7; H, 3.7; N, 13.5.

*[Yb_2_(ipapm)_2_(NO_3_)_4_] (**2**).* The colorless plate-like crystals of **2** were isolated by a filtration of the reaction acetonitrile solution containing Yb(NO_3_)_3_·6H_2_O (61 mg, 0.1 mmol) and Rad (47.8 mg, 0.222 mmol), which reduced volume through slow evaporation for two days. Yield: 4.5 mg. Anal. calcd. (%) for C_28_H_28_N_10_O_16_Yb_2_: C, 30.38; H, 2.55; N, 12.66. Found: C, 30.4; H, 2.5; N, 12.7.

*[Ce_2_(ipapm)_2_(NO_3_)_4_(EtOAc)_2_]·0.5EtOAc (**3**)* The colorless thin crystals of **3** were isolated, by filtration, from the saturated ethylacetate reaction acetonitrile solution (during one night) containing Ce(NO_3_)_3_·6H_2_O (43.5 mg, 0.1 mmol) and Rad (60 mg, 0.222 mmol). The drying of the compound resulted in an unsolvated complex *[Ce_2_(ipapm)_2_(NO_3_)_4_(EtOAc)_2_].* Yield: 5.5 mg. Anal. calcd. (%) for C_38_H_48_Ce_2_N_10_O_21_: C, 36.19; H, 3.8; N, 11.10. Found: C, 36.4; H, 3.7; N, 11.3.

*4,4-dimethyl-2,2-bis(2-pyridyl)oxazolidine* (**4**). The title compound was synthesized according to a known procedure [9]. The colorless single crystals of **4** were grown using a slow evaporation solution of the bulk product in acetylacetate.

The yellow-orange single crystals of *4,4-dimethyl-2,2-bis(pyridin-2-yl)-1,3-oxazolidine-3-oxyl (Rad)* (**5**) were obtained through the slow evaporation of the radical toluene solution.

*4,4-dimethyl-3-[(2-methyl-2-nitro-propoxy)-bis(2-pyridyl)methoxy]-2,2-bis(2-pyridyl)oxazolidine* (**6**). A small amount of the crystals of **6** was prepared through the further evaporation of the mother liquor remaining after the synthesis of (**5**).

## 4. Conclusions and Perspectives

During the synthesis of diradical complexes from the hydrated salts of the lanthanides, one mononuclear complex and two binuclear complexes containing diamagnetic ligands were obtained. These ligands were the products of the transformation of the oxazolidine radical functionalized by the pyridyl groups in the coordination sphere of the lanthanide ion, wherein the processes of simultaneous disproportionation, hydrolysis of 4,4-dimethyl-2,2-bis(pyridin-2-yl)-1,3-oxazolidine-3-oxyl, and their condensation in the solution proceed differently than in the absence of metal ions. This is confirmed by the structurally characterized product of the radical transformation in a toluene solution.

Since the obtained binuclear complexes are of particular interest due to their magnetic and photophysical properties, in the near future, additional research will be carried out to define the synthetic conditions necessary for obtaining these compounds larger quantities.

## Data Availability

The complete crystallographic data for **1**–**6** have been deposited with the Cambridge Crystallographic Data Centre under the reference numbers CCDC 2131650-2131655, respectively. These data can be obtained, free of charge, from CCDC via www.ccdc.cam.ac.uk/structures (accessed on 29 December 2021).

## Supplementary Materials

The following are available online, Figure S1: Ln^3+^ ion supported catalytic hydrolysis transformation of the Rad into tetradentate ligand, *ipapm*.; Table S1: Crystal data and structure refinement for compounds **1**–**3**; Table S2: Crystal data and structure refinement for compounds **4**–**6**; Table S3: Selected geometric parameters of **1**; Table S4: Selected geometric parameters for an independent structural unit of [Yb_2_(*ipapm*)_2_(NO_3_)_4_] (**2)**; Table S5: Selected geometric parameters for an independent structural unit of dimer [Ce_2_(*ipapm*)_2_(NO_3_)_4_(EtOAc)_2_] (**3**); Table S6: Selected geometric parameters of **4**; Table S7: Selected geometric parameters of **5**; Table S8: Selected geometric parameters of **6**.
