# Supplementary material for: Oxazolidine Nitroxide Transformation in a Coordination Sphere of the Ln3+ Ions
Source: Molecules. 2022 Mar 1;27(5):1626. doi: 10.3390/molecules27051626 (PMC8911955; doi:10.3390/molecules27051626)
Supplement: Supplementary file 1 [file molecules-27-01626-s001.zip › molecules-1560447-supplementary.pdf]

Supporting Information on

Oxazolidine Nitroxide Transformation in a Coordination Sphere of the Ln^{3+} Ions

by Philippe Rey, Anton I. Smoletsev, and Kira E. Vostrikova*

Nikolaev Institute of Inorganic Chemistry, Siberian Branch, Russian Academy of Sciences

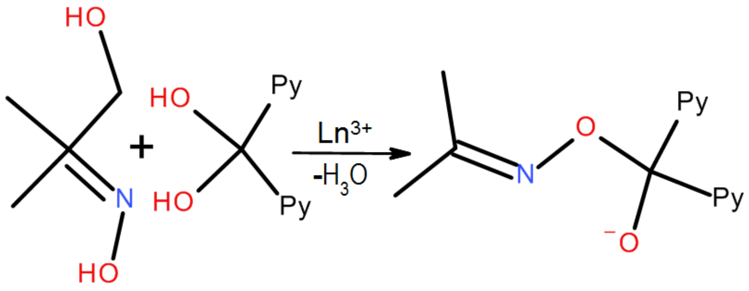

Figure S1. Ln^{3+} ion supported catalytic hydrolysis transformation of the Rad into tetradentate ligand, *ipapm*.

Table S1. Crystal data and structure refinement for compounds **1–3.**

Identification code	**1**	**2**	**3**
Empirical formula	$C_{28}H_{29}Cl_2DyN_6O_4$	$C_{28}H_{28}N_{10}O_{16}Yb_2$	$C_{40}H_{52}Ce_2N_{10}O_{22}$
Formula weight	746.97	1106.68	1305.15
Temperature/K	150(2)	150(2)	150(2)
Space group	$P\bar{1}$	$P\bar{1}$	$P\bar{1}$
a/Å	10.1300(4)	9.5039(3)	10.7445(3)
b/Å	10.1774(5)	10.2339(3)	11.2134(3)
c/Å	15.2401(7)	10.7319(3)	12.0491(4)
α/°	92.624(2)	113.7970(10)	116.3640(10)
β/°	108.139(2)	103.6100(10)	90.3170(10)
γ/°	99.099(2)	101.4350(10)	1292.87(7)
Volume/Å^3	1466.76(12)	876.34(5)	1292.87(7)
Z	2	1	1
ρ_{calc}/g cm^{-3}	1.691	2.097	1.676
μ/mm^{-1}	2.774	5.392	1.826
F(000)	742	534	654
Crystal size/mm^3	0.10 × 0.10 × 0.08	0.10 × 0.08 × 0.04	0.15 × 0.12 × 0.04
θ range/°	2.037 to 28.690	3.743 to 30.532	2.039 to 30.532
h, k, l indices range	$-13 \le h \le 13$, $-13 \le k \le 13$, $-20 \le l \le 20$	$-13 \le h \le 13$, $-14 \le k \le 14$, $-15 \le l \le 15$	$-15 \le h \le 15$, $-14 \le k \le 16$, $-17 \le l \le 15$
Reflections collected	24900	17673	22391
Independent reflections	7410 [R_{int} = 0.0558]	5286 [R_{int} = 0.0364]	7381 [R_{int} = 0.0454]
Observed reflections	6435 [$I > 2\sigma(I)$]	4766 [$I > 2\sigma(I)$]	6257 [$I > 2\sigma(I)$]
Refined parameters/ restraints	375/0	255/0	349/27
Goodness-of-fit on F^2	1.070	1.087	1.167
$R[F^2 > 2\sigma(F^2)]$	R_1 = 0.0428, wR_2 = 0.1046	R_1 = 0.0353, wR_2 = 0.0719	R_1 = 0.0445, wR_2 = 0.0773
$R(F^2)$ (all data)	R_1 = 0.0519, wR_2 = 0.1124	R_1 = 0.0411, wR_2 = 0.0765	R_1 = 0.0592, wR_2 = 0.0999
Largest diff. peak/hole/eÅ^{-3}	2.338/-0.968	2.391/-1.478	1.147/-1.313

[Dy(RadH)(*hbpm*)Cl_2] CH_3CN (**1**), [Yb_2(*ipapm*)$_2$(NO_3)$_4$] (**2**), [Ce_2(*ipapm*)$_2$(NO_3)$_4$(EtOAc)$_2$]·0.5EtOAc (**3**)

Table S2. Crystal data and structure refinement for compounds **4–6**.

Identification code	**4**	**5**	**6**
Empirical formula	$C_{15}H_{17}N_3O$	$C_{15}H_{16}N_3O_2$	$C_{40.5}H_{44}N_6O_5$
Formula weight	255.31	270.31	694.81
Temperature/K	150(2)	293(2)	300(2)
Space group	*Cc*	*Cc*	$P3_2$
a/Å	14.3765(8)	14.9436(8)	18.0065(7)
b/Å	7.9972(5)	8.2469(4)	
c/Å	12.1337(6)	12.1134(5)	9.0454(5)
β/°	105.412(2)	112.917(2)	
Volume/Å^3	1344.87(13)	1375.01(12)	2539.9(2)
Z	4	4	3
ρ_{calc}/g cm^{-3}	1.261	1.306	1.363
μ/mm^{-1}	0.082	0.089	0.091
F(000)	544	572	1107
Crystal size/mm^3	0.15 × 0.15 × 0.10	0.25 × 0.15 × 0.10	0.17 × 0.12 × 0.05
θ range/°	2.939 to 30.516	4.782 to 26.360	1.306 to 25.685
h, k, l indices range	$-20 \le h \le 19$, $-11 \le k \le 9$, $-17 \le l \le 15$	$-18 \le h \le 18$, $-10 \le \mathrm{k} \le 10$, $-15 \le l \le 14$	$-17 \le h \le 21$, $-21 \le k \le 20$, $-11 \le l \le 11$
Reflections collected	8838	5446	20995
Independent reflections	3600 [R_{int} = 0.0325]	2684 [R_{int} = 0.0315]	6405 [R_{int} = 0.0391]
Observed reflections	3346 [$I > 2\sigma(I)$]	2382 [$I > 2\sigma(I)$]	4958 [$I > 2\sigma(I)$]
Refined parameters/ restraints	177/2	252/15	374/1
Goodness-of-fit on F^2	1.052	1.082	0.957
$R[F^2 > 2\sigma(F^2)]$	R_1 = 0.0482, wR_2 = 0.1313	R_1 = 0.0358, wR_2 = 0.0766	R_1 = 0.0423, wR_2 = 0.0990
$R(F^2)$ (all data)	R_1 = 0.0514, wR_2 = 0.1352	R_1 = 0.0426, wR_2 = 0.0801	R_1 = 0.0575, wR_2 = 0.1045
Largest diff. peak/hole/eÅ^{-3}	0.344/-0.246	0.122/-0.108	0.161/-0.134

Table S3. Selected geometric parameters of **1.**

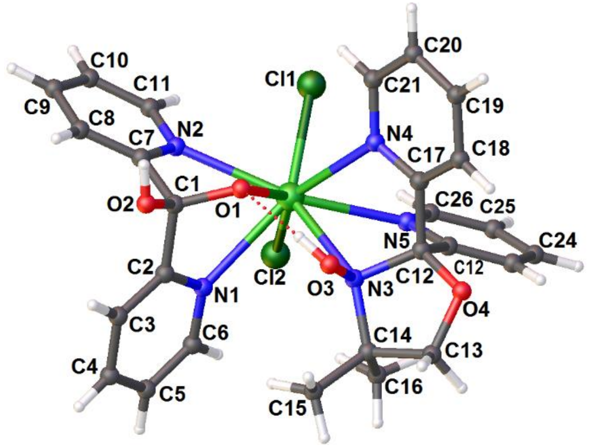

Bond	Length, Å	Bond	Length, Å	Angle	°
Dy1—O1	2.270 (3)	C4—C5	1.379 (8)	O1—Dy1—N5	131.94 (2)
Dy1—N5	2.529 (4)	N5—C22	1.343 (6)	O1—Dy1—N2	65.82 (11)
Dy1—N2	2.550 (4)	N5—C26	1.357 (6)	N5—Dy1—N2	159.50 (3)
Dy1—N1	2.599 (4)	C5—C6	1.377 (7)	O1—Dy1—N1	66.83 (1)
Dy1—N4	2.614 (4)	N6—C28	1.146 (9)	N5—Dy1—N1	122.98 (2)
Dy1—Cl1	2.6264 (12)	C7—C8	1.385 (6)	N2—Dy1—N1	70.42 (2)
Dy1—Cl2	2.6445 (12)	C8—C9	1.391 (7)	O1—Dy1—N4	73.48 (1)
Dy1—N3	2.715 (4)	C9—C10	1.384 (7)	N5—Dy1—N4	67.68 (2)
O1—C1	1.393 (5)	C10—C11	1.378 (7)	N2—Dy1—N4	117.01 (2)
N1—C2	1.342 (6)	C12—C17	1.517 (6)	N1—Dy1—N4	131.35 (2)
N1—C6	1.353 (6)	C12—C22	1.533 (7)	O1—Dy1—Cl1	115.24 (8)
C1—O2	1.390 (5)	C13—C14	1.545 (8)	N5—Dy1—Cl1	84.05 (10)
C1—C7	1.533 (6)	C14—C15	1.519 (8)	N2—Dy1—Cl1	77.75 (9)
C1—C2	1.535 (6)	C14—C16	1.531 (7)	N1—Dy1—Cl1	143.66 (9)
N2—C11	1.339 (6)	C17—C18	1.374 (7)	N4—Dy1—Cl1	78.82 (9)
N2—C7	1.339 (6)	C18—C19	1.378 (7)	O1—Dy1—Cl2	140.80 (9)
C2—C3	1.386 (7)	C19—C20	1.382 (7)	N5—Dy1—Cl2	77.49 (10)
O3—N3	1.448 (5)	C20—C21	1.388 (7)	N2—Dy1—Cl2	92.91 (9)
N3—C12	1.487 (6)	C22—C23	1.385 (7)	N1—Dy1—Cl2	75.25 (8)
N3—C14	1.522 (6)	C23—C24	1.384 (8)	N4—Dy1—Cl2	144.13 (9)
C3—C4	1.387 (7)	C24—C25	1.358 (8)	Cl1—Dy1—Cl2	89.61 (4)
O4—C12	1.414 (5)	C25—C26	1.378 (7)	O1—Dy1—N3	72.78 (11)
O4—C13	1.433 (7)	C27—C28	1.428 (9)	N5—Dy1—N3	65.39 (12)
N4—C21	1.344 (6)	C9—C10	1.384 (7)	N2—Dy1—N3	135.10 (2)
N4—C17	1.351 (6)			N1—Dy1—N3	78.10 (2)

Table S4. Selected geometric parameters of a monomeric moiety of [Yb_2(*ipapm*)$_2$(NO_3)$_4$] (**2**).

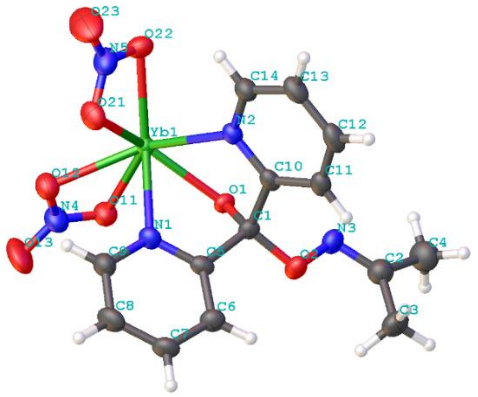

Bond	Length, Å	Angle	°
Yb1—O1^{i}	2.208 (3)	O1^{i}—Yb1—O1	68.93 (12)
Yb1—O1	2.265 (3)	O1^{i}—Yb1—O21	141.60 (12)
Yb1—O21	2.371 (3)	O1—Yb1—O21	138.24 (12)
Yb1—O11	2.392 (3)	O1^{i}—Yb1—O11	84.23 (12)
Yb1—O12	2.397 (3)	O1—Yb1—O11	77.19 (11)
Yb1—O22	2.408 (3)	O21—Yb1—O11	122.70 (12)
Yb1—N1	2.493 (4)	O1^{i}—Yb1—O12	126.10 (12)
Yb1—N2	2.557 (4)	O1—Yb1—O12	120.54 (12)
Yb1—N3^{i}	2.647 (4)	O21—Yb1—O12	69.92 (12)
Yb1—Yb1^{i}	3.6877 (4)	O11—Yb1—O12	52.99 (12)
O1—C1	1.382 (5)	O1^{i}—Yb1—O22	88.73 (11)
N1—C5	1.329 (6)	O1—Yb1—O22	130.96 (11)
N1—C9	1.363 (6)	O21—Yb1—O22	53.29 (12)
C1—O2	1.404 (5)	O11—Yb1—O22	145.52 (12)
C1—C5	1.525 (6)	O12—Yb1—O22	108.03 (12)
C1—C10	1.538 (6)	O1^{i}—Yb1—N1	136.04 (12)
O2—N3	1.433 (5)	O1—Yb1—N1	67.49 (11)
N2—C14	1.337 (6)	O21—Yb1—N1	79.24 (13)
N2—C10	1.344 (6)	O11—Yb1—N1	80.97 (13)
C2—N3	1.283 (7)	O12—Yb1—N1	73.96 (13)
C2—C4	1.470 (8)	O22—Yb1—N1	124.86 (12)
C2—C3	1.493 (7)	O1^{i}—Yb1—N2	94.60 (11)
N4—O13	1.221 (5)	O1—Yb1—N2	66.53 (11)
N4—O11	1.242 (5)	O21—Yb1—N2	80.53 (12)
N4—O12	1.273 (5)	O11—Yb1—N2	141.28 (12)
N5—O23	1.209 (6)	O12—Yb1—N2	139.11 (12)
N5—O21	1.261 (6)	O22—Yb1—N2	72.84 (12)
N5—O22	1.271 (6)	N1—Yb1—N2	73.23 (12)
C5—C6	1.394 (6)	O1^{i}—Yb1—N3^{i}	64.09 (12)
C6—C7	1.376 (7)	O1—Yb1—N3^{i}	129.30 (12)
C7—C8	1.388 (7)	O21—Yb1—N3^{i}	91.92 (13)
C8—C9	1.377 (7)	O11—Yb1—N3^{i}	80.20 (13)
C10—C11	1.390 (6)	O12—Yb1—N3^{i}	76.34 (13)
C11—C12	1.387 (7)	O22—Yb1—N3^{i}	66.45 (13)
C12—C13	1.383 (8)	N1—Yb1—N3^{i}	150.27 (13)
C13—C14	1.383 (7)	N2—Yb1—N3^{i}	133.67 (12)

Symmetry code: (i) −*x*+1, −*y*+1, −*z*+1.

Table S5. Selected geometric parameters of a monomeric moiety of [Ce_2(*ipapm*)$_2$(NO_3)$_4$(EtOAc)$_2$] (**3**)

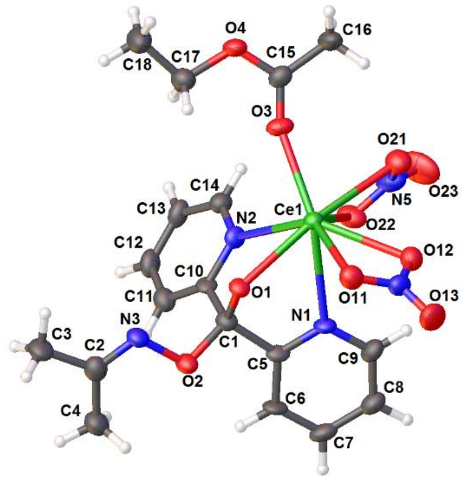

Bond	Length, Å	Angle	°
Ce1—O1^{i}	2.400 (3)	O1^{i}—Ce1—O1	65.83 (11)
Ce1—O1	2.449 (3)	O1^{i}—Ce1—O3	76.03 (10)
Ce1—O3	2.530 (3)	O1—Ce1—O3	114.95 (11)
Ce1—O12	2.576 (3)	O1^{i}—Ce1—O12	124.28 (10)
Ce1—O22	2.590 (3)	O1—Ce1—O12	113.13 (10)
Ce1—O11	2.607 (3)	O3—Ce1—O12	131.90 (11)
Ce1—N2	2.684 (3)	O1^{i}—Ce1—O22	151.32 (11)
Ce1—N1	2.727 (3)	O1—Ce1—O22	117.52 (10)
Ce1—O21	2.733 (4)	O3—Ce1—O22	77.37 (11)
Ce1—N3^{i}	2.813 (4)	O12—Ce1—O22	82.07 (12)
Ce1—Ce1^{i}	4.0702 (5)	O1^{i}—Ce1—O11	80.43 (11)
O1—C1	1.364 (5)	O1—Ce1—O11	76.03 (10)
N1—C5	1.317 (6)	O3—Ce1—O11	145.87 (11)
N1—C9	1.355 (6)	O12—Ce1—O11	49.20 (11)
C1—O2	1.443 (5)	O22—Ce1—O11	128.21 (12)
C1—C10	1.526 (6)	O1^{i}—Ce1—N2	94.62 (10)
C1—C5	1.537 (6)	O1—Ce1—N2	61.62 (11)
O2—N3	1.449 (5)	O3—Ce1—N2	71.86 (11)
N2—C14	1.334 (6)	O12—Ce1—N2	136.11 (11)
N2—C10	1.344 (5)	O22—Ce1—N2	66.84 (12)
C2—N3	1.281 (6)	O11—Ce1—N2	135.01 (11)
C2—C3	1.478 (7)	O1^{i}—Ce1—N1	126.22 (10)
C2—C4	1.507 (7)	O1—Ce1—N1	61.93 (10)
O3—C15	1.214 (6)	O3—Ce1—N1	138.35 (11)
O4—C15	1.319 (5)	O12—Ce1—N1	69.07 (11)
O4—C17	1.461 (6)	O22—Ce1—N1	70.37 (11)
N4—O13	1.226 (5)	O11—Ce1—N1	75.76 (11)
N4—O11	1.263 (5)	N2—Ce1—N1	71.62 (11)
N4—O12	1.266 (5)	O1^{i}—Ce1—O21	128.54 (10)
O5—C19	1.185 (8)	O1—Ce1—O21	164.72 (10)
N5—O23	1.221 (5)	O3—Ce1—O21	68.98 (11)
N5—O21	1.257 (5)	O12—Ce1—O21	65.03 (11)
N5—O22	1.270 (6)	O22—Ce1—O21	47.68 (11)
C5—C6	1.400 (6)	O11—Ce1—O21	109.38 (11)

C6—C7	1.375 (7)	N2—Ce1—O21	108.32 (11)
C7—C8	1.391 (7)	N1—Ce1—O21	104.83 (11)
C8—C9	1.368 (7)	O1^{i}—Ce1—N3^{i}	62.57 (10)
C11—C12	1.376 (7)	O1—Ce1—N3^{i}	123.46 (10)
C12—C13	1.381 (7)	O3—Ce1—N3^{i}	73.07 (11)
C12—H12	0.9500	O12—Ce1—N3^{i}	79.89 (11)
C13—C14	1.385 (6)	O22—Ce1—N3^{i}	118.74 (11)
C15—C16	1.483 (7)	O11—Ce1—N3^{i}	74.27 (11)
C17—C18	1.480 (7)	N2—Ce1—N3^{i}	141.89 (12)
C19—C20	1.565 (9)	N1—Ce1—N3^{i}	146.38 (12)
C21—C22	1.400 (8)	O21—Ce1—N3^{i}	71.72 (11)
Symmetry codes: (i) −*x*+1, −*y*+1, −*z*+1; (ii) −*x*, −*y*+1, −*z*.			

Table S6. Selected geometric parameters of **4.**

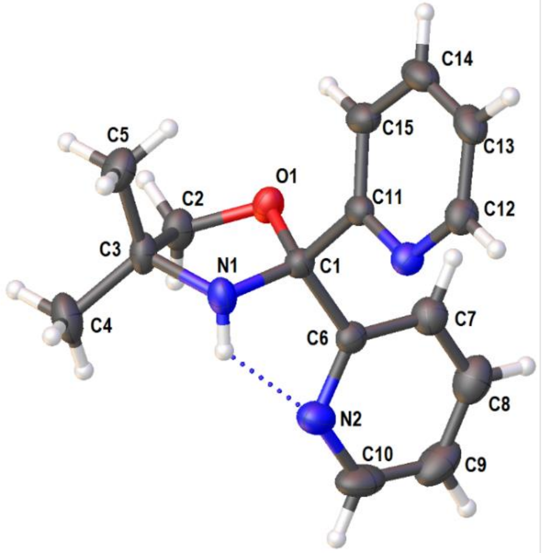

Bond	Length, Å	Angle	°
Dy1—O1	2.270 (3)	O1—Dy1—N5	131.94 (12)
Dy1—N5	2.529 (4)	O1—Dy1—N2	65.82 (11)
Dy1—N2	2.550 (4)	N5—Dy1—N2	159.50 (13)
Dy1—N1	2.599 (4)	O1—Dy1—N1	66.83 (11)
Dy1—N4	2.614 (4)	N5—Dy1—N1	122.98 (12)
Dy1—Cl1	2.6264 (12)	N2—Dy1—N1	70.42 (12)
Dy1—Cl2	2.6445 (12)	O1—Dy1—N4	73.48 (11)
Dy1—N3	2.715 (4)	N5—Dy1—N4	67.68 (12)
O1—C1	1.393 (5)	N2—Dy1—N4	117.01 (12)
N1—C2	1.342 (6)	N1—Dy1—N4	131.35 (12)
N1—C6	1.353 (6)	O1—Dy1—Cl1	115.24 (8)
C1—O2	1.390 (5)	N5—Dy1—Cl1	84.05 (10)
C1—C7	1.533 (6)	N2—Dy1—Cl1	77.75 (9)
C1—C2	1.535 (6)	N1—Dy1—Cl1	143.66 (9)
N2—C11	1.339 (6)	N4—Dy1—Cl1	78.82 (9)
N2—C7	1.339 (6)	O1—Dy1—Cl2	140.80 (9)
C2—C3	1.386 (7)	N5—Dy1—Cl2	77.49 (10)
O3—N3	1.448 (5)	N2—Dy1—Cl2	92.91 (9)

O3—H3A	0.8400	N1—Dy1—Cl2	75.25 (8)
N3—C12	1.487 (6)	N4—Dy1—Cl2	144.13 (9)
N3—C14	1.522 (6)	Cl1—Dy1—Cl2	89.61 (4)
C3—C4	1.387 (7)	O1—Dy1—N3	72.78 (11)
O4—C12	1.414 (5)	N5—Dy1—N3	65.39 (12)
O4—C13	1.433 (7)	N2—Dy1—N3	135.10 (12)
N4—C21	1.344 (6)	N1—Dy1—N3	78.10 (12)
N4—C17	1.351 (6)	N4—Dy1—N3	63.86 (12)
C4—C5	1.379 (8)	Cl1—Dy1—N3	138.20 (9)
N5—C22	1.343 (6)	Cl2—Dy1—N3	109.35 (8)
N5—C26	1.357 (6)	C1—O1—Dy1	114.4 (2)
C5—C6	1.377 (7)	C2—N1—C6	117.3 (4)
N6—C28	1.146 (9)	C2—N1—Dy1	111.9 (3)
C7—C8	1.385 (6)	C6—N1—Dy1	130.7 (3)
C8—C9	1.391 (7)	O2—C1—O1	113.6 (4)
C9—C10	1.384 (7)	O2—C1—C7	113.4 (4)
C10—C11	1.378 (7)	O1—C1—C7	107.3 (4)
C12—C17	1.517 (6)	O2—C1—C2	106.5 (4)
C12—C22	1.533 (7)	O1—C1—C2	109.6 (3)
C13—C14	1.545 (8)	C7—C1—C2	106.2 (4)
C14—C15	1.519 (8)	C1—O2—H2	109.5
C14—C16	1.531 (7)	C11—N2—C7	117.8 (4)
C17—C18	1.374 (7)	C2—N1—Dy1	111.9 (3)
C18—C19	1.378 (7)	C6—N1—Dy1	130.7 (3)
C19—C20	1.382 (7)	C11—N2—Dy1	128.6 (3)
C20—C21	1.388 (7)	C7—N2—Dy1	113.4 (3)
C22—C23	1.385 (7)	O3—N3—Dy1	106.0 (2)
C23—C24	1.384 (8)	C12—N3—Dy1	101.5 (3)
C24—C25	1.358 (8)	C14—N3—Dy1	134.3 (3)
C25—C26	1.378 (7)	C21—N4—Dy1	123.9 (3)
C27—C28	1.428 (9)	C17—N4—Dy1	118.5 (3)

Table S7. Selected geometric parameters of **5.**

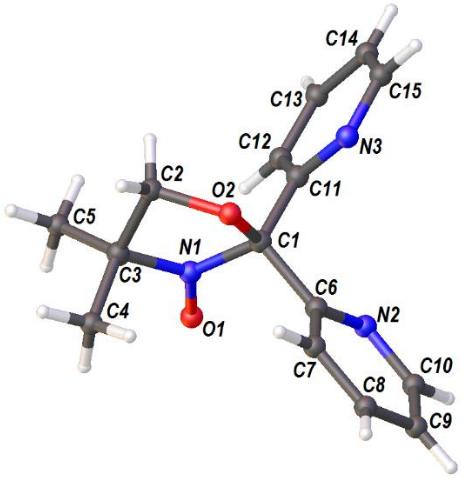

Bond	Length, Å	Angle	°
O1—N1	1.252 (4)	C1—O2—C2	106.9 (3)
O2—C1	1.422 (5)	O1—N1—C1	123.9 (3)
O2—C2	1.444 (5)	O1—N1—C3	123.0 (5)
N1—C1	1.476 (5)	C1—N1—C3	112.7 (5)
N1—C3	1.488 (8)	C10—N2—C6	116.4 (2)
N2—C10	1.332 (4)	C11—N3—C15	116.4 (3)
N2—C6	1.337 (3)	O2—C1—N1	101.8 (3)
N3—C11	1.331 (3)	O2—C1—C6	111.2 (3)
N3—C15	1.344 (4)	N1—C1—C6	108.3 (3)
C1—C6	1.524 (5)	O2—C1—C11	112.3 (3)
C1—C11	1.538 (5)	N1—C1—C11	111.2 (3)
C2—C3	1.527 (12)	C6—C1—C11	111.5 (3)
C3—C5	1.515 (8)	O2—C2—C3	104.5 (4)
C3—C4	1.521 (7)	N1—C3—C5	108.5 (6)
C6—C7	1.383 (3)	N1—C3—C4	109.5 (6)
C7—C8	1.380 (4)	C5—C3—C4	113.0 (6)
C8—C9	1.372 (4)	N1—C3—C2	97.8 (5)
C9—C10	1.378 (4)	C5—C3—C2	114.6 (6)
C11—C12	1.365 (3)	C4—C3—C2	112.2 (6)
C12—C13	1.378 (4)	N1—C3—C5	108.5 (6)
C13—C14	1.350 (5)	N1—C3—C4	109.5 (6)
C14—C15	1.369 (5)	C5—C3—C4	113.0 (6)
C8—C9—C10	118.1 (3)	N2—C6—C7	123.5 (2)
N2—C10—C9	124.4 (2)	N2—C6—C1	118.0 (2)
		C7—C6—C1	118.5 (3)
		C8—C7—C6	118.4 (2)
		N3—C11—C12	123.5 (2)
		N3—C11—C1	110.1 (2)
		C12—C11—C1	126.4 (3)
		C11—C12—C13	118.7 (3)
		C14—C13—C12	119.1 (3)

		C13—C14—C15	119.0 (3)
		N3—C11—C12	123.5 (2)
		N3—C11—C1	110.1 (2)
		C12—C11—C1	126.4 (3)
		C11—C12—C13	118.7 (3)
		N3—C11—C12	123.5 (2)
		C14—C13—C12	119.1 (3)
		C13—C14—C15	119.0 (3)
		N3—C15—C14	123.3 (3)

Table S8. Selected parameters geometric of **6.**

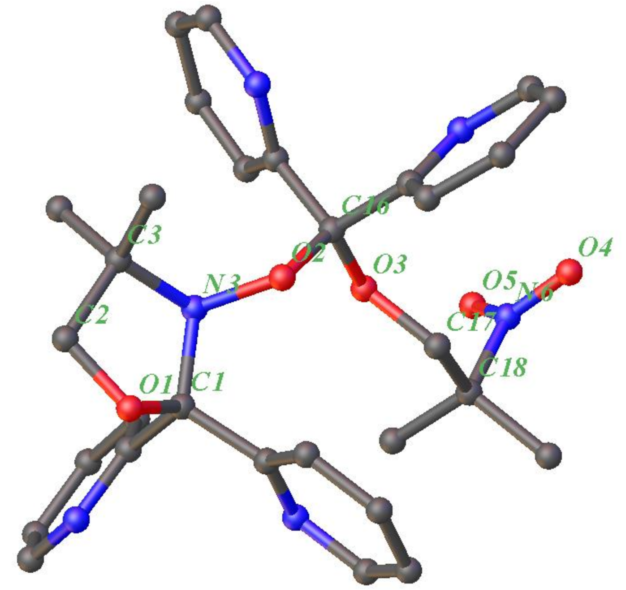

Bond	Length, Å	Angle	°
O3—C16	1.406 (3)	O4—N6—O5	124.3 (4)
O3—C17	1.410 (3)	O4—N6—C18	118.0 (3)
O2—C16	1.432 (3)	O5—N6—C18	117.7 (4)
O2—N3	1.455 (3)	C16—O2—N3	111.84 (19)
O1—C2	1.405 (4)	O2—N3—C1	109.2 (2)
O1—C1	1.419 (4)	O2—N3—C3	109.5 (2)
N3—C1	1.498 (4)	C1—N3—C3	104.0 (2)
N3—C3	1.536 (4)	C16—O3—C17	117.7 (2)
N6—O4	1.202 (4)	C16—O2—N3	111.84 (19)
N6—O5	1.213 (4)	O2—N3—C1	109.2 (2)
N6—C18	1.534 (5)	O2—N3—C3	109.5 (2)
